# miR-432-5p Inhibits the Ferroptosis in Cardiomyocytes Induced by Hypoxia/Reoxygenation via Activating Nrf2/SLC7A11 Axis by Degrading Keap1

**DOI:** 10.1155/2023/1293200

**Published:** 2023-10-03

**Authors:** Wei Geng, Shaohua Yan, Xinyue Li, Qiumei Liu, Xuefei Zhang, Xinshun Gu, Xiang Tian, Yunfa Jiang

**Affiliations:** ^1^Department of Cardiology, Baoding No. 1 Central Hospital, Baoding City, Hebei, China; ^2^Department of Cardiology, The Second Hospital of Hebei Medical University, Shijiazhuang City, Hebei, China

## Abstract

Early reperfusion into the myocardium after ischemia causes myocardial ischemia–reperfusion (I/R) injury and ferroptosis was involved. Ischemia activates the expression of a series of oxidative stress genes and their downstream regulatory genes, including ferroptosis-related genes such as nuclear factor E2-related factor 2 (Nrf2), glutathione peroxidase 4 (GPX4), and SLC7A11. This study adopted primary cardiomyocytes and I/R in rats to evaluate the ferroptosis and changing of Nrf2-SLC7A11/heme oxygenase-1 (HO-1) *in vitro* and *in vivo*. Online analysis tools were used to predict the possible target Kelch-like ECH-associated protein 1 (Keap1) of miR-432-5p. The mimic of miR-432-5p plasmid was constructed to verify the effect of miR-432-5p on ferroptosis. We found that hypoxia/reoxygenation (H/R) in cardiomyocytes and I/R in rats induced lipid peroxidation and ferroptosis in cardiomyocytes. The activation of the Nrf2-SLC7A11/HO-1 pathway protects cardiomyocytes from ferroptosis. Downregulation of miR-432-5p has been confirmed in H/R cardiomyocytes (*in vitro*) and cardiomyocytes in myocardial infarction rats (*in vivo*). Upregulation of miR-432-5p inhibited ferroptosis of cardiomyocytes induced by RAS-selective lethal 3 (RSL3), an inhibitor of GPX4 and ferroptosis inducer through decreasing the binding protein of Nrf2, Keap1, which was confirmed by bioinformatics and mutation assay. Knockdown Nrf2 attenuates the protection effect of miR-432-5p on H/R cardiomyocytes. Intravenous delivery of liposome carriers of miR-432-5p remarkably ameliorated cardiomyocyte impairment in the I/R animal model. In conclusion, miR-432-5p inhibits the ferroptosis in cardiomyocytes induced by H/R by activating Nrf2/SLC7A11 axis by degrading Keap1 and is a potential drug target for clinical myocardial infarction treatment.

## 1. Introduction

Myocardial infarction (MI) is one of the cardiovascular diseases threatening human health. It is also the main cause of unexpected death and heart failure. Due to the myocardial cell injury and death caused by the interruption of local blood supply to the heart, patients may have a poor prognosis of disability [1]. The early recanalization of occluded coronary arteries can preserve the potential necrosis of myocardium or reduce the area of myocardial necrosis. However, early reperfusion is often accompanied by the decline of myocardial systolic and diastolic function, the occurrence of reperfusion arrhythmia, the changes in myocardial energy metabolism, and the changes in myocardial structure, that is, myocardial ischemia–reperfusion (I/R) injury [2].

Clarifying the pathogenesis of myocardial infarction is helpful to seek new interventions, further reducing myocardial ischemia injury and improving the prognosis of patients [3]. When myocardial infarction occurs, myocardial cells are irreversibly damaged and necrotic due to hypoxia and decreased ATP supply [4]. The necrotic cells activate the immune system after releasing their debris, resulting in a severe inflammatory response. The release of inflammatory mediators starts the body's repair of damaged tissues. On the other hand, continuous inflammation will lead to matrix degradation and cardiomyocyte apoptosis [5], and excessive reactive oxygen species (ROS) will cause damaged cell structure, and even cell death, which leads to the sequelae of myocardial infarction.

Ischemia activates the expression of a series of oxidative stress genes and their downstream regulatory genes [6, 7]. The nuclear factor E2-related factor 2 (Nrf2)/Kelch-like ECH-associated protein 1 (Keap1)/heme oxygenase-1 (HO-1) is an important endogenous antioxidant pathway, especially the Nrf2, it acts as a “switch” to regulate the oxidation reaction [8]. The Nrf2 has been proven to act as one of the critical ferroptosis genes in many diseases [9, 10], including myocardial infarction [11]. Hypoxia induces the production of ROS and Nrf2, and they express in both sustained and intermittent conditions. Keap1 exists in the cytoplasm through binding with Nrf2 and mediates the inhibition of Nrf2. When oxidative stress increases *in vivo*, Nrf2 dissociates from Keap1 protein and transfers to the nucleus, it binds with antioxidant response element (ARE), and then induces the increased expression of HO-1, regulates the activity of a series of reactive elements, including lipid peroxidation products, malondialdehyde (MDA), glutathione (GSH), superoxide dismutase (SOD), and other antioxidant stress factors [12].

Ferroptosis is a typical oxidative stress-related death pathway. Recent studies have shown that ferroptosis plays an important role in the regulation of oxidative stress and inflammatory responses [13, 14]. Iron chelating agents and antioxidants could inhibit ferroptosis [15]. The cystine-glutamate exchanger (xCT) or SLC7A11 provides intracellular cyst(e)ine to produce glutathione. Glutathione peroxidase 4 (GPX4) is the key regulator of ferroptosis [16]. Sims et al. [17] have indicated that xCT has neuroprotective in hypoxic preconditioning. In a mice model with acute lung injury, knocking down Nrf2 notably decreased the expression of SLC7A11 and HO-1 [18]. This shows that Nrf2 may regulate SLC7A11 through the same pathway during ischemia, but their regulatory role in myocardial infarction needs further clarified.

The miRNAs mediate posttranscriptional regulation of gene expression, and they also integrally take part in the ferroptosis regulatory network [19]. The miR-432-5p has been largely reported involving the regulation process of different tumors [20–22]. Akdemir et al. [23] demonstrated that miR-432 induces Nrf2 stabilization by targeting Keap1 directly in esophageal squamous cell carcinoma cells while rare reports mentioned its roles in MI. In this study, we explored expression change of miR-432-5p in hypoxia-treated primary cardiomyocytes and cardiomyocytes in hypoxic animal, and further clarify its role in ferroptosis through regulating Nrf2/Keap1 pathway.

## 2. Methods

### 2.1. Primary Culture of Neonatal Rat Ventricular

By following the methods mentioned by Maass and Buvoli [24], newborn (within 48 hr of birth) Sprague Dawley (SD) rats were decapitated by small scissors, disinfected by 75% alcohol twice, and the heart was squeezed to remove the left ventricle and immersed into precooled phosphate-buffered saline (PBS) solution quickly. The heart was cut into pieces and digested by a mixture containing 0.05% trypsin and 0.05% collagenase for 5 min; the supernatant was discarded after precipitation. After repeated three times, the mixture was centrifuged at 1,500 rpm for 5 min; the supernatant was removed, and Dulbecco's modified Eagle medium/nutrient mixture F-12 (DMEM-F12) medium (Sigma–Aldrich) containing 15% fetal bovine serum (FBS) was added to culture cells for 2 hr. The culture medium containing cell suspension was changed to another culture dish after 1 hr using differential adherent method to remove other types of cells. Then, 0.1 mmol/L 5-bromodeoxyuridine (BrdU, Selleck) was added to inhibit the growth of fibroblasts. The medium was replaced without 5-Brdu after 48 hr of culture and then cultured for 24 hr until rhythmic beating myocardial cells could be seen under a microscope.

### 2.2. Oxygen Deprivation and Reoxygenation Model of Cells

The primary cardiomyocytes were cultured in a DMEM medium containing 10% FBS and 5-BrdU (200 *μ*mol/L) under 37°C and in an incubator containing 5% CO_2_. *In vitro* hypoxia/reoxygenation (H/R) model was established, briefly, to simulate anoxic conditions; logarithmic growing cells were inoculated in six-well plates with a concentration of 1 × 10^6^ cells/well, and cultured for 4 and 6 hr in an incubator supplied with 95% N_2_ and 5% CO_2_ at 37°C. Cells were then put into the incubator with 37°C, 95% air, and 5% CO_2_ for 6 hr.

### 2.3. Cell Transfection and Experimental Grouping

The cells were transfected with Lipofectamine 2000 (#11668027; Thermo Fisher Scientific, USA) according to the manufacturer's instruction [25]. Hypoxia reoxygenation treatment was carried out 48 hr after transfection. In this study, primary cardiomyocytes were transfected with the following sequence: the mimic of miR-432-5p expression plasmid, miR-432-5p mimic control (miR-432-5p-NC), Keap1-wild type (Keap1-WT), Keap1-mutant sequence (Keap1-Mut), Nrf2 invalid interference sequence (nc-Nrf2), and interference sequences of Nrf2 (si-Nrf2). The pcDNA3.1(+) vector (#VT1010; YouBio Tec. Inc., Changsha, China) was used for miR-432-5p expression construction, and genes were inserted into the KpnI and XbaI enzyme restriction site. All sequences were designed and synthesized by Gene Pharma (Shanghai, China). The sequence information was listed in Table 1. To measure the related gene expressions, quantitative reverse transcription-polymerase chain reaction (qRT-PCR) was adopted. For quantification of gene expression, the 2-^*ΔΔ*Ct^ method was used. U6 and glyceraldehyde-3-phosphate dehydrogenase (GAPDH) were used as references.

### 2.4. Cell Viability

The logarithmic primary cardiomyocytes were inoculated into 96-well plates with a concentration of 2 × 10^3^ cells/well. After transfection, and H/R treatment mentioned above was followed in cells. Ten microliter of CCK8 solution (#C0042, Beyotime, China) was added to each well. Cells were cultured in the incubator for 4 hr, and the absorbance value at 450 nm was measured by a microplate reader (Multiskan™ FC, Thermo Fisher, USA) to calculate the cell survival rate. Cell viability rate (%) = (absorbance value of treatment group/absorbance value of control group) ×100%.

### 2.5. Measurement of MDA, SOD, GSH/GSSG Ratio, and LDH in Cells

Cardiomyocytes received different treatments were collected. Cell lysate (#P0013, Beyotime, China) was added to lyse cells on ice for 30 min according to the manufacturer's instruction. The lysed cells were centrifuged at 4°C with 12,000 rpm/min for 15 min, and the supernatant was collected. MDA, SOD, GSH/glutathione disulfide (GSSG), and lactate dehydrogenase (LDH) were measured by the commercial kits (#S0131S, #S0101S, #S0053, #C0017, Beyotime, China), respectively. For the LDH assay, background control was subtracted to make more accuracy of cytotoxicity calculation.

### 2.6. miR-432-5p Expression in Patients with Myocardial Infarction from Human Database

The Gene Expression Omnibus (GEO) database (http://www.ncbi.nlm.nih.gov/geo/) was adopted to screen potential candidate miRNA in patients with acute myocardial infarction. Screened keywords include “microRNA or noncoding RNA, patients, and acute myocardial infarction.” The noncoding RNA expression profiles of GSE24548 and GSE31568 were downloaded and analyzed finally. Both datasets contained noncoding RNA profiling, which derived from plasma samples from acute myocardial infarction patients and matched healthy control. miR-432-5p expression level from each subject was isolated and analyzed using GEO2R (http://www.ncbi.nlm.nih.gov/geo/geo2r/), which is a web tool that can analyze almost GEO data.

### 2.7. Dual-Luciferase Reporter Gene Assay

The rat cardiomyocyte H9c2 cells were obtained from the cell bank of the Chinese Academy of Sciences (Shanghai, China). Cells were cultured in a DMEM medium containing 10% FBS and 1% streptomycin (#11966025, Gibco, UK) under 37°C and in an incubator containing 5% CO_2_. A Dual Luciferase Assay Kit (#E1910, Promega) was used and performed in H9c2 cells. The 3′-UTR sequence of Keap1 containing the miR-432-5p binding site was amplified by PCR. The binding site was edited to construct Keap1 wild-type plasmid (Keap1-WT) and mutant plasmid (Keap1-Mut), respectively. The Keap1-WT or Keap1-Mut was cotransfected with miR-432-5p mimic or miR-432-5p-NC plasmid into cells by Lipofectamine™ 2000 Transfection Reagent (#11668027; Thermo Fisher). After 24 hr of transfection, the cells were collected and lysed. One hundred microliter LARII solution and 100 *μ*L Stop-Glo solution were added in 20 *μ*L of cell lysate and the detect firefly and sea kidney luciferase activity was measured.

### 2.8. I/R Rat Model

Healthy male SD rats, weight 250–300 g, age 16–20 weeks, were purchased from the Institute of Laboratory Animal Sciences, Chinese Academy of Medical Sciences (CAMS) and Peking Union Medical College (PUMC), China. The rats were placed and anesthetized with 80 mg/kg of pentobarbital following Wu et al.'s report [26]. The chest at the left fourth intercostal space was cut, and the heart was squeezed out to ligate the anterior descending branch of the left coronary artery. The ligation was performed at the lower edge between the left atrial appendage and the pulmonary artery cone with a 0/5 noninvasive suture. Rats were considered to have acute myocardial ischemia injury when the electrocardiogram (ECG) shows widening or increasing of R or ST-segment elevation measured by a single-channel electrocardiograph (CONTEC Medical system, Qinghuangdao, China). Meanwhile, the color of the anterior wall of the left ventricle and the apex of the heart was darkened and the pulsation was weakened. The ligation was not performed in rats from the sham group. The anterior descending branch was ligated for 30 min and reperfusion was resumed for 4 hr by anesthetization with pentobarbital. During the operation, the rats' limbs were connected by needle electrodes and the ECG was recorded with standard II lead. Six rats per group were recruited in sham and I/R model. All animal experiments were conducted in strict accordance with the guidance on treating experimental animals issued by the Ministry of Science and Technology of China. The Ethics Committee of Baoding No.1 Central Hospital approved related protocols (Approval number HB20210236).

### 2.9. Measurement of Myocardial Infarction Size

After the 4-hr reperfusion period, 1 mL of saturated potassium chloride solution (25%) was injected into the right ventricular chamber to stop the heart from beating. The heart is then rapidly excised, embedded in 2% agarose PBS, and then sectioned in 1–2-mm slices. The extent of infarct size was evaluated by the Alcian Blue-2,3,5-triphenyltetrazolium chloride (TTC) method. The infarction size was calculated using ImageJ 1.31 software (NIH, USA). The outer edge of the necrotic scarred myocardium (a) and the perimeter of the normal myocardium (b) were measured and the myocardial infarction size = a/(a + b) × %.

### 2.10. Measurement of MDA, SOD, and GSH/GSSG in MI Rats

Blood was collected through the abdominal aorta immediately after reperfusion. The blood was centrifuged at 1,000 *g* at 4°C for 15 min, and the serum was stored in the −80°C refrigerator. The serum level of MDA, SOD, and GSH/GSSG was tested by the related commercial kit (#S0131S, #S0101S, #S0053, Beyotime, China) according to the instructions and previous publications [27, 28].

### 2.11. Histological Analysis in Cardiac Tissues

Following the methods mentioned by Li et al. [29], histological analysis was performed in resected cardiac tissues. Briefly, hearts were fixed with 4% formaldehyde, embedded in paraffin, and prepared into 5-*μ*m sections. After undergoing some dewaxing and transparency treatment, the hematoxylin & eosin (H&E) staining was performed and 4-hydroxynonenal (4-HNE) antibody (#ARG70025, Arigo Biolaboratories, China) was added to sections for histochemistry staining.

### 2.12. MicroRNA Liposome Treatment

Healthy male SD rats, weight 250–300 g, age 16–20 weeks, were purchased from the Institute of Laboratory Animal Sciences, CAMS and PUMC, China. All the animals were divided into three groups (*N* = 8): sham, I/R receiving miR-NC, and I/R receiving miR-432-5p mimic. Liposome, a commonly used miRNA delivery method, was used to encapsulate miR-432-5p in our study. The miR-432-5p mimic oligonucleotide was prepared into 0.2% mass concentration and mixed with 40 mg/L cationic liposomes to form 0.1% oligonucleotide liposomes and 0.1% miRNA-free blank liposomes. Preventive administration was carried out 1 day earlier on the day of MI induction, and miR-432-5p mimic (final concentration 5 *μ*g/g body weight) was injected into a caudal vein [30], once a day for the two consecutive days, and once week for the following 3 weeks.

### 2.13. Western Blot Analysis

Western blot was performed according to the previous protocols [31, 32]. Cardiomyocytes or homogenized rat heart tissues were collected and treated by cell lysis buffer (#P0013, Beyotime, China) to extract cellular proteins. BCA Kit (#P0010S, Beyotime, China) was used to detect the protein concentration. Because Nrf2 plays its role in nuclei, the nuclear protein was isolated separately by nuclear protein extraction kit (Thermo Fisher, CA, USA). A total of 30 *μ*g of protein samples were loaded for sodium dodecyl sulfate polyacrylamide gel electrophoresis to separate proteins. After the membrane was transferred and sealed, the primary antibodies for Keap1 (#10503-2-AP, 1 : 2000, Proteintech), Nrf2 (#16396-1-AP, 1 : 1000, Proteintech), SLC7A11 (#26864-1-AP, 1 : 1000, Proteintech), GPX4 (#MA5-35089, 1 : 1000, Invitrogen), UCP3 (#AB3046, 1 : 1000, Sigma-Aldrich), and PTGS2 (ab179800, 1 : 1000, Abcam) were added and then were incubated with a suitable secondary antibody at room temperature for 1 hr. Proteins were developed by enhanced chemiluminescence and imaged by the gel imaging system. The ImageJ 1.31 software (NIH, USA) was used to analyze the gray value of each protein band.

### 2.14. Statistical Analysis

GraphPad Prism 8.0 (GraphPad Software, Inc., La Jolla, CA, USA) was used for statistical analysis. All data were expressed by mean ± standard deviation (SD). The Student's *t*-test was used to compare the two groups with normally distributed data; the rank sum test was used to compare nonnormal distributed data between two groups. The one-way analysis of variance (ANOVA) was used for multigroup comparison. The difference was statistically significant with *p* < 0.05.

## 3. Results

### 3.1. Hypoxia/Reoxygenation Induces Lipid Peroxidation and Ferroptosis in Cardiomyocytes

**Figure 1:**
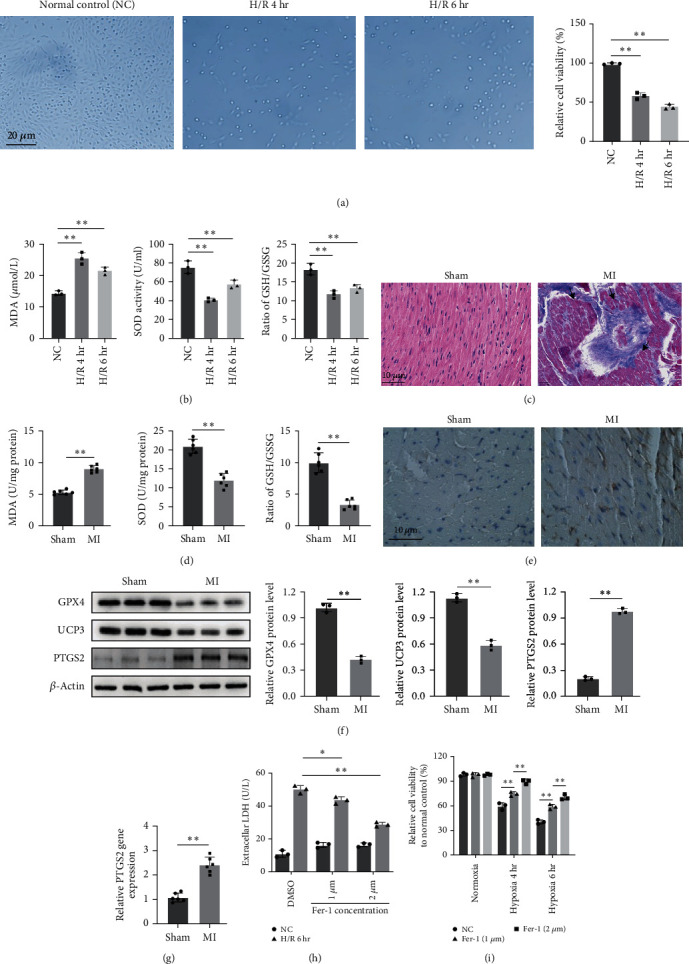
The Fe metabolism is involved in the process of cell hypoxia/reoxygenation (H/R) and ischemia/reperfusion (I/R): (a) the cell morphology (magnification was 100x) and viability of cardiomyocytes under H/R 4 hr and H/R 6 hr decreased compared to normal control by LDH assay, *N* = 3; (b) H/R leads to increased MDA level, decreased SOD activity, and decreased ratio of GSH/GSSG in cardiomyocytes; (c) representative image of normal heart and I/R heart section stained by H&E (*N* = 6, magnification was 200x); MI section demonstrates unclear myofibril structure, reticular fibrosis can be seen in myocardium, and lipofuscin can be seen in the cytoplasm of myocardial cells (black arrow); (d) *in vivo* analysis demonstrated that I/R also caused increased MDA, decreased SOD, and the ratio of GSH/GSSG, *N* = 6; (e) the immunohistochemistry of 4-HNE in cardiac tissues. I/R induces obviously elevated 4-HNE, one of the products of lipid peroxidation (magnification was 200x); (f) GPX4 and UCP3 protein level decreased after I/R; (g) I/R increased mRNA and protein expression of ferroptosis biomarker PTGS2; the extracellular LDH level decreased under H/R conditions and (h) cell viability (i) had been recovered.  ^*∗*^*p* < 0.05,  ^*∗∗*^*p* < 0.01; *N* = 6.

The viability of cardiomyocytes was measured at two time points, 4 and 6 hr after *in vitro* H/R treatment. Results showed that H/R decreased the viability of cardiomyocytes *in vitro* and causes partial cell deaths (Figure 1(a)), which is accompanied by a significant increase in MDA level and significantly decreased SOD activity and ratio of GSH/GSSG (Figure 1(b)). The *in vivo* MI animal model demonstrated that the I/R also causes serious cardiomyocyte damage by pathological observation (H&E staining, Figure 1(c)), which demonstrated as disappeared myocardial nuclei and striations, unclear myofibril structure, reticular fibrosis can be seen in myocardium, and lipofuscin can be seen in the cytoplasm of myocardial cells (black arrow). The measurement in MI hearts also demonstrated that I/R led to increased MDA, decreased SOD activity, and ratio of GSH/GSSG, which suggested that ferroptosis might occur (Figure 1(d)). The 4-HNE staining also indicates obviously elevated 4-HNE, one of the end products of lipid peroxidation, in cardiac tissues (Figure 1(e)). The western blot analysis of GPX4 protein, a catalyzer decreasing cellular lipid oxidative stress, reduced, as well as uncoupling protein 3 (UCP3), a cardioprotective marker of mitochondrial damage [33], decreased in MI heart (Figure 1(f)). qRT-PCR analysis and western blot also demonstrated that I/R boosted the levels of ferroptosis biomarkers PTGS2 in rat-impaired cardiac tissues (Figures 1(f) and 1(g)). Moreover, after cardiomyocytes were treated with ferrostatin-1 (Fer-1), a ferroptosis inhibitor, the extracellular LDH level decreased under H/R conditions (Figure 1(h)) and cell viability (Figure 1(i)) had been recovered. These results demonstrated that ferroptosis was involved in the process of cardiomyocyte injury caused by H/R (*in vitro*) and by I/R (*in vivo*).

### 3.2. The Activation of the Nrf2-SLC7A11/HO-1 Pathway Protects Cardiomyocytes from Ferroptosis

**Figure 2:**
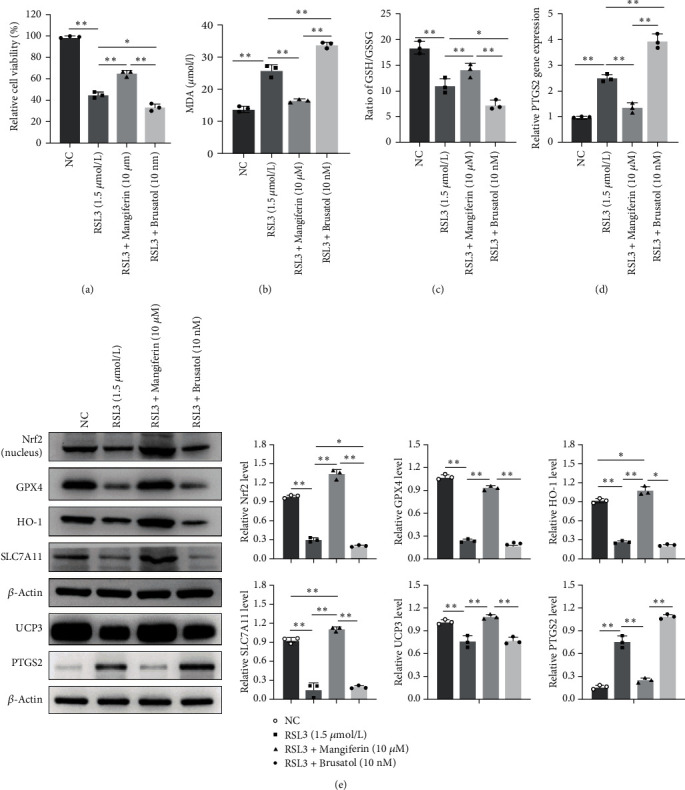
The activation of the Nrf2/SLC7A11/GPX4/HO-1 pathway protects cardiomyocytes from ferroptosis: (a) the ferroptosis inducer RSL3 (1.5 *μ*M) reduced the viability of cardiomyocytes significantly; (b–d) RSL3-induced ferroptosis caused increased MDA and PTGS2 levels and the downregulated ratio of GSH/GSSG; (e) representative western blot image of protein expressions. Pretreatment of Nrf2 activator, mangiferin, suppressed apoptosis of cardiomyocytes induced by RSL3; increased Nrf2, GPX4, SLC7A11, HO-1, and UCP3 expression, while the Nrf2 inhibitor, brusatol (10 nM), aggravates the ferroptosis by decreasing Nrf2, SLC7A11, HO-1, and UCP3 expressions.  ^*∗*^*p* < 0.05,  ^*∗∗*^*p* < 0.01; *N* = 3.

To investigate the role of Nrf2-SLC7A11/HO-1 in ferroptosis of cardiomyocytes, the *in vitro* ferroptosis was induced by RSL3, a ferroptosis inducer. RSL3 (1.5 *μ*M) reduced the viability of cardiomyocytes significantly (Figure 2(a)). As we expected, treatment with RSL3 increased MDA and PTGS2 levels and the downregulated ratio of GSH/GSSG (Figure 2(b)–2(d)), which suggested that ferroptosis occurred. By Western blot, we noticed that Nrf2, GPX4, SLC7A11, and HO-1 were all decreased by RSL3 (Figure 2(e)). Consistent with *in vivo* analysis result, the PTGS2 also increased in ferroptosis introduced by RSL3, while UCP3 showed reversed trend (Figure 2(e)). Pretreatment of Nrf2 activator, mangiferin [34], significantly reversed ferroptosis-related characteristics in cardiomyocytes induced by RSL3, such as lower viability, higher MDA and PTGS2 levels, and reduced ratio of GSH/GSSG (Figure 2(a)–2(d)). Western blot analysis also demonstrated that expression of Nrf2, GPX4, SLC7A11, HO-1, and UCP3 increased after being treated with mangiferin (Figure 2(e)). Oppositely, the Nrf2 inhibitor, brusatol (10 nM), aggravates the lipid peroxidation and ferroptosis induced by RSL3 through decreasing Nrf2, GPX4, SLC7A11, HO-1, and UCP3 and increasing PTGS2 expressions (Figure 2(e)). These results demonstrated that upregulation of Nrf2 protects cardiomyocytes from ferroptosis.

### 3.3. miRNA-432-5p Attenuates Ferroptosis in Cardiomyocytes

**Figure 3:**
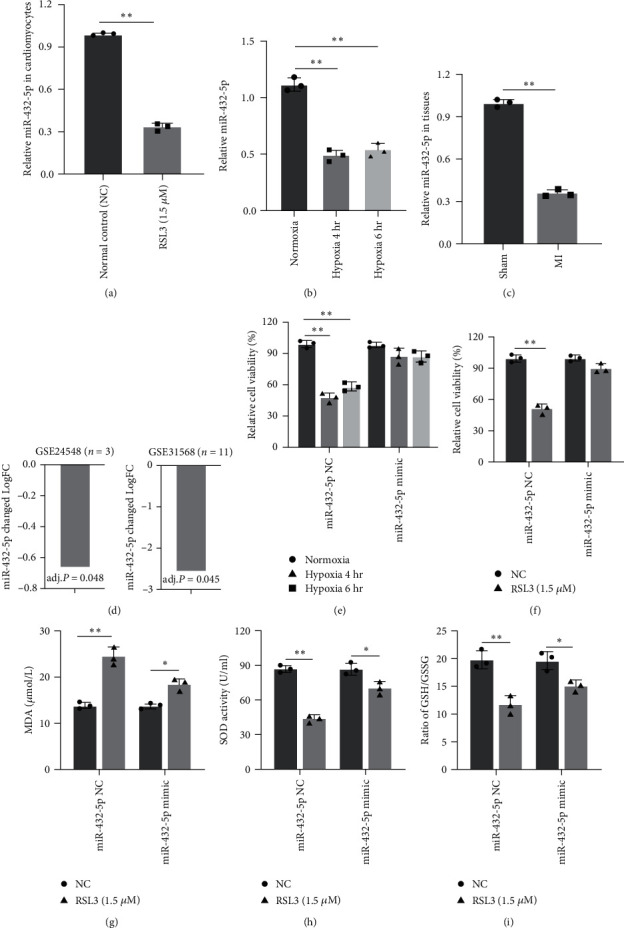
miRNA-432-5p attenuates ferroptosis in cardiomyocytes under H/R: (a and b) miR-432-5p was significantly decreased in the cardiomyocytes after RSL3 stimulation (a) and under H/R (b); (c) miR-432-5p decreased in MI tissue compared with control tissue (sham); (d) expressions of miR-432-5p in two GSE datasets, GSE24548 and GSE31568, that were also downregulated in patients with acute MI compared with normal control; (e–i) to measure the effect of miRNA-432-5p in ferroptosis caused by RSL3, plasmid-harboring miRNA-432-5p was transfected to cardiomyocytes under H/R. Upregulation of miRNA-432-5p improves cell viability, decreases MDA level, increases SOD, and GSH/GSSG ratio of cardiomyocytes after RSL3 stimulation.  ^*∗*^*p* < 0.05,  ^*∗∗*^*p* < 0.01; *N* = 3.

RT-PCR showed that miR-432-5p was significantly decreased in the cardiomyocytes after *in vitro* H/R and under RSL3 treatment, as well as in myocardial tissue of MI rats (Figure 3(a)–3(c)). In addition, expressions from two GSE datasets, GSE24548, and GSE31568, also demonstrate that miRNA-432-5p is downregulated in patients with acute MI compared with healthy control (Figure 3(d)). These results indicated that miRNA-432-5p might be involved in the ferroptosis process caused by I/R. To further investigate its function in ferroptosis, miRNA-432-5p was transfected and the viability of cardiomyocytes under H/R and RSL3 challenge with miRNA-432-5p mimic (30 nM) was significantly elevated compared with NC control (Figures 3(e) and 3(f)). Results also showed increased miRNA-432-5p by mimic decreased MDA level, increased SOD activity, and GSH/GSSG ratio of cardiomyocytes under RSL3 challenge (Figure 3(g)–3(i)).

### 3.4. miR-432-5p Ameliorates the Ferroptosis by Activating Nrf2 through Degrading Keap1

**Figure 4:**
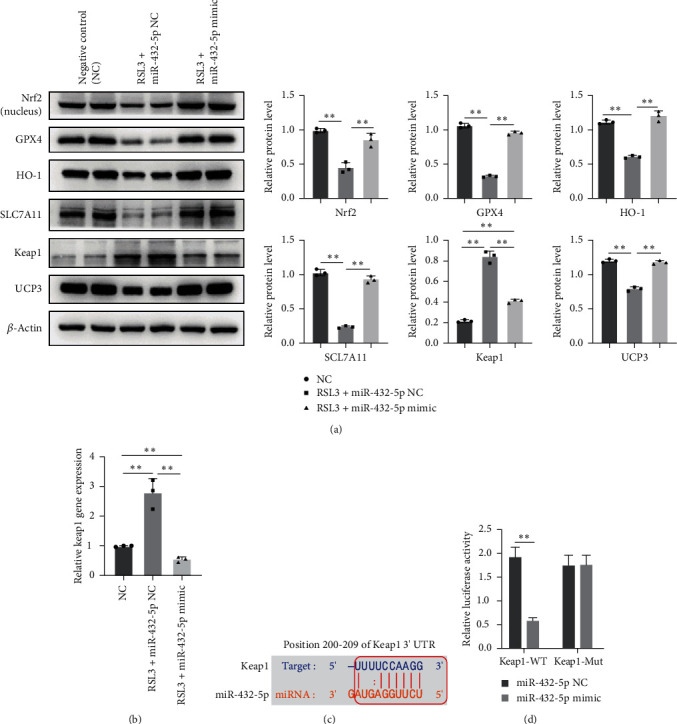
miR-432-5p activates Nrf2 by degrading Keap1 under RSL3 stimulation: (a) representative image of protein expressions of Nrf2, GPX4, HO-1, SLC7A11, and Keap1 in cardiomyocytes under RSL3 stimulation. Upregulation of miR-432-5p leads to the increased expression of Nrf2, GPX4, HO-1, and SLC7A11 while decreased Keap1; (b) miRNA-432-5p mimic decreased Keap1 expression at mRNA level; (c) the binding site of miRNA-432-5p with Keap1 predicted by TargetScan (https://www.targetscan.org/) online software; (d) dual-luciferase reporter gene assay was used to analyze binding efficacy of miR-432-5p to Keap1 gene, and the luciferase activity decreased significantly in cells containing Keap1-WT sequence, which demonstrated miR-432-5p mimic decreased Keap1 gene expression.  ^*∗∗*^*p* < 0.01; *N* = 3.

Western blot analysis revealed that upregulation of miR-432-5p leads to the increased expression of Nrf2, which was reduced by RSL3 challenge (Figure 4(a)). The expression of GPX4, SLC7A11, and HO-1 increased along with the Nrf2 while Keap1 decreased (Figure 4(a)). miR-432-5p also reversed the UCP3 protein level reduced by RSL3 (Figure 4(a)). These results suggested that the protective effect of miRNA-432-5p against ferroptosis in cardiomyocytes might due to enhancing the activation of Nrf2 signaling pathway. Further, we analyzed the mRNA expression of Keap1. Results demonstrated that RSL3 challenge also caused increasing mRNA expression of Keap1, while miRNA-432-5p mimics decreased Keap1 expression (Figure 4(b)). Bioinformatical analysis online indicated that miR-432-5p could bind Keap1 (Figure 4(c)), and the binding sites were predicted by TargetScan (https://www.targetscan.org/) gene prediction software. The dual-luciferase reporter gene analysis showed that the luciferase activity was significantly decreased after cotransfection of miR-432-5p mimic plasmid with pGL3 3′-UTR of Keap1-WT reporter vector, but not its mutation counterpart (*p* < 0.01 vs. Keap1-Mut reporter vector) (Figure 4(d)).

### 3.5. Knockdown of Nrf2 Abolished the Protection Effect of miRNA-432-5p against Ferroptosis in Cardiomyocytes

**Figure 5:**
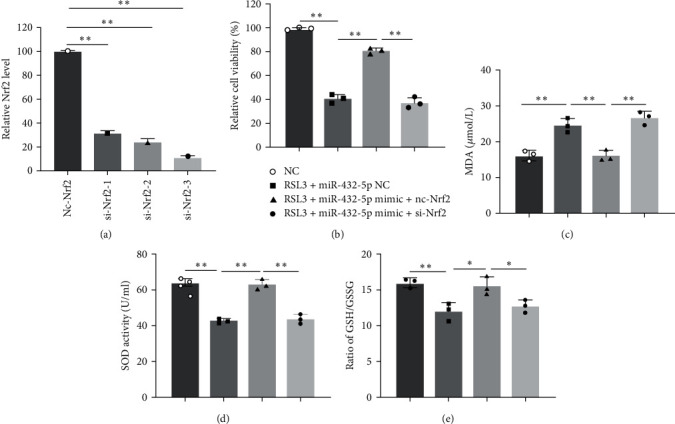
Knockdown of Nrf2 abolished the attenuation of miRNA-432-5p on ferroptosis in cardiomyocytes: (a) the interference efficiency of three designed sequences; (b–e) knocking down Nrf2 significantly abolished protection effect of miRNA-432-5p against ferroptosis caused by RSL3 in cell viability (b), MDA levels (c), SOD activity (d), and GSH/GSSG ratio (e)  ^*∗*^*p* < 0.05,  ^*∗∗*^*p* < 0.01; *N* = 3.

To verify that the protection effect of miRNA-432-5p against ferroptosis in cardiomyocytes was dependent with Nrf2, we knocked down Nrf2 in cardiomyocytes with siRNA with the highest interference efficiency (Figure 5(a)). Knocking down Nrf2 partially abolished protection effect of miRNA-432-5p against ferroptosis caused by RSL3, which was demonstrated by decreased cell viability, SOD activity, GSH/GSSG ratio, and increased MDA levels after introducing miRNA-432-5p mimic (Figure 5(b)–5(e)). These results showed that ferroptosis-resistant effect of miRNA-432-5p was dependent with Nrf2 signaling pathway.

### 3.6. miRNA-432-5p Attenuates MI in Rats through Upregulation of Nrf2 In Vivo

**Figure 6:**
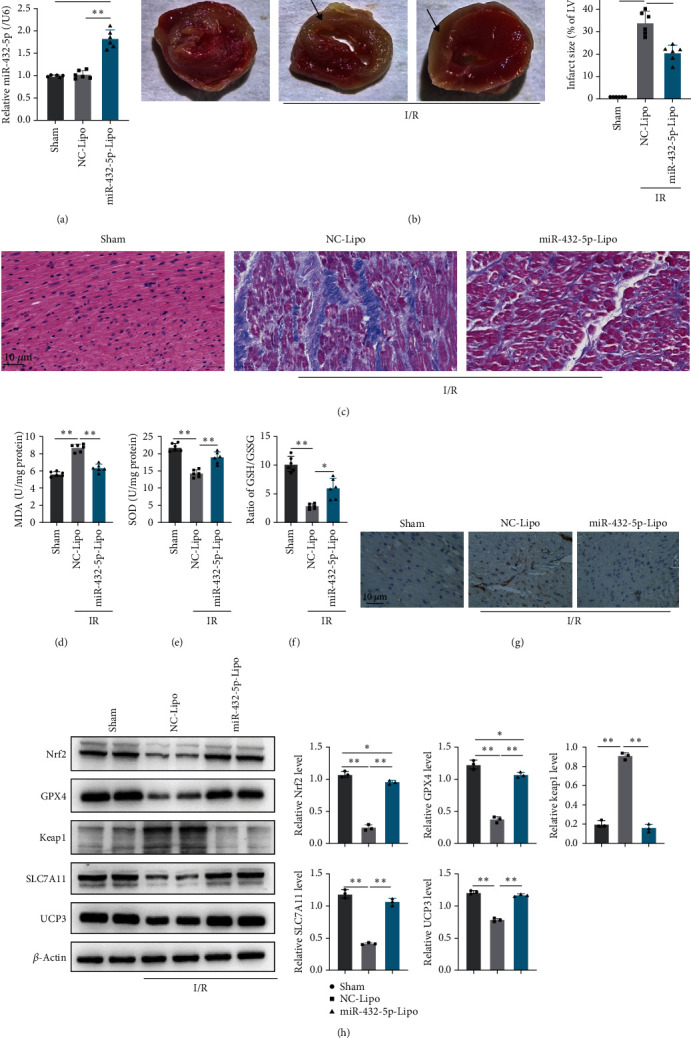
miRNA-432-5p attenuates I/R caused myocardial injury in rats through upregulation of Nrf2 *in vivo:* (a) the level of exogenous miR-432-5p in the heart tissue; (b) representative image of TTC stained heart in the sham group, I/R rats received miR-432-5p negative control (NC-Lipo), and miR-432-5p mimic (miR-432-5p-Lipo), arrows referred to the damage area; (c) H&E staining was performed in heart sections, miR-432-5p mimic alleviated the injury of infarction in rat heart (magnification was 200x); (d–f) miR-432-5p alleviates the injury of infarction in rat heart through decreasing MDA, increases SOD, and the increases ratio of GSH/GSSG in vivo; (g) miR-432-5p mimic attenuates I/R caused lipid peroxidation in cardiac tissues. Positive immunohistochemistry of 4-HNE in miR-432-5p-Lipo decreases in I/R rats (magnification was 200x); (h) representative western blot image of protein expressions of Nrf2, GPX4, Keap1, and SLC7A11 in rats from the sham group, I/R rats received miR-432-5p negative control and miR-432-5p mimic. miR-432-5p caused decreased expression of Keap1 and increased expression of Nrf2, GPX4, and SLC7A11 in the I/R myocardium.  ^*∗*^*p* < 0.05,  ^*∗∗*^*p* < 0.01; *N* = 6.

To verify the therapy potential of miR-432-5p against cardiomyocyte injury caused by I/R *in vivo*, the MI rats were established by I/R surgery and cationic liposomes carrying miR-432-5p mimic were administrated through vein tail 24 hr prior I/R. The level of exogenous miR-432-5p in the heart tissue is shown in Figure 6(a). Compared with NC-Lipo group, partial improved heart function could be observed in miR-432-5p-Lipo group, which demonstrated as decreased infarction size (Figure 6(b)), improved heart rate, and left ventricular developed pressure (Table 2). The miR-432-5p mimic alleviated the injury of infarction in rat heart (Figures 6(b) and 6(c)) by TTC and H&E staining, which was manifested by reducing the rupture of partial myocardial cells, decreased the formation of myocardial lysosomes, or decreased the necrosis of myocardial contraction bands. The miR-432-5p liposome decreased MDA, increased SOD, and the ratio of GSH/GSSG in I/R rats (Figure 6(d)–6(f)). Immunohistochemical positive 4-HNE proteins under light microscope showed a obviously decreased 4-HNE in miR-432-5p liposome-treated cardiac tissues (Figure 6(g)). In sham group, there was a small amount of Keap1 protein expression in cardiac tissues and the expression of GPX4, Nrf2, and SLC7A11 was high. GPX4, Nrf2, and SLC7A11 were significantly reduced, while Keap1 was significantly increased in the hearts of rats receiving empty liposomes. Compared with the empty liposome group, miR-432-5p liposome enhanced the expression of GPX4, Nrf2, and SLC7A11 protein in the heart, while Keap1 expression was inhibited by western blot (Figure 6(h)). The decreased UCP3 was also reversed by miR-532-5p mimics treatment (Figure 6(h)). These results demonstrated that miR-432-5p causes decreased expression of Keap1 and increased expression of GPX4, Nrf2, and SLC7A11 in I/R heart when compared to rats received liposome packaging.

## 4. Discussion

**Figure 7:**
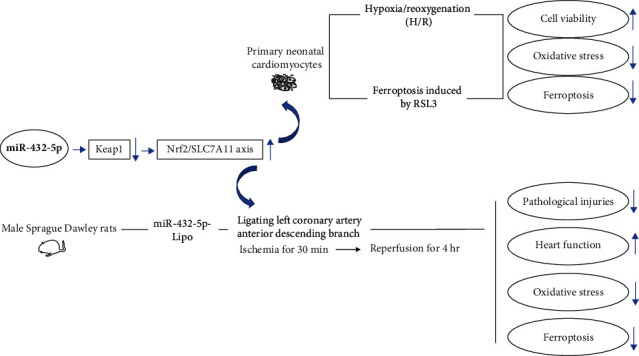
The summary of possible mechanisms of myocardial protection of miR-432-5p via activating Nrf2/SLC7A11 axis by degrading Keap1.

In this study, we analyzed the regulation of Nrf2 to myocardial cell injury in acute myocardial infarction. We found that ferroptosis was involved in the injury process of myocardial cells after hypoxia and rats with myocardial infarction. The activation of the Nrf2-GPX4/SLC7A11/HO-1 pathway protects cardiomyocytes from ferroptosis. We also found that the regulation of Nrf2 on ferroptosis in cardiomyocytes is mainly or partially achieved by miR-432-5p binding Keap1. The possible mechanisms of myocardial protection of miR-432-5p are summarized in Figure 7.

Hypoxia/ischemia could lead to the death of myocardial cells. As an antagonistic response of the body, upregulation of Nrf2 can slow down the cell damage caused by hypoxia. Myocardial I/R refers to the pathological process in which ischemic myocardium recovers normal perfusion when partially or completely blocked coronary arteries are reopened, but its tissue damage shows progressive aggravation. Classical I/R injury has many pathophysiological processes, such as ion accumulation, mitochondrial membrane damage, ROS formation, lipid oxidation, endothelial dysfunction, platelet aggregation, immune activation, apoptosis, and autophagy [35, 36]. These pathological processes lead to changes in the structure and function of myocardial tissue. Baba et al. [37] confirmed for the first time that ferroptosis occurs in cardiomyocytes and noncardiomyocytes during I/R *in vivo*. Inhibiting ferroptosis of cardiomyocytes during I/R can effectively reduce infarct area and markers of myocardial injury and improve cardiac remodeling. The mechanisms of RSL3-mediated ferroptosis are not clear, and RSL3 can inhibit the antioxidant system [38]. Our research shows that Nrf2 inactivation might be one of possible mechanisms of RSL3-mediated ferroptosis and the specific mechanisms may need further exploration and verification.

Nrf2 is one of the most important transcription factors regulating oxidative stress *in vivo*. Existing studies have found that Nrf2 plays an important neuroprotective role in brain I/R injury [39]. Nrf2 activator can upregulate the expression of antioxidant protein and scavenge ROS. After activation, Nrf2 reduces the maturation and expression of inflammatory factors such as interleukin-1*β* (IL-1*β*), tumor necrosis factor-*α* (TNF-*α*), and IL-18 to inhibit the inflammatory response after cerebral I/R injury [40, 41]. Under physiological conditions, Nrf2 exists in the cytoplasm and Keap1 protein anchors Nrf2 to the cytoskeleton of actin, preventing Nrf2 from entering the nucleus to exert its transcriptional activity. Under stress conditions, Nrf2 is activated and enters the nucleus through the shuttle mechanism. Nrf2 entering the nucleus combines with small Maf protein to form heterodimers, recognize ARE, and activate downstream antioxidant genes [42]. In this study, we found that in MI rats, the expression level of Nrf2 protein decreased and the expression level of Keap1 protein increased. At the same time, serum MDA level increased, while SOD level and the ratio of GSH/GSSG decreased. After exogenously increasing the expression of miR-432-5p, the level of Nrf2 protein increased while Keap1 decreased significantly, indicating that the Nrf2-Keap1 pathway was activated by miR-432-5p to attenuate ferroptosis caused by ischemia/hypoxia.

Ferroptosis is different from traditional apoptosis, necrosis, and autophagy in appearance, biochemistry, and gene profiles. The morphological characteristics of ferroptosis are mitochondrial shrinkage, increased membrane density, and mitochondrial cristae reduction or disappearance. The molecular characteristics are intracellular iron overload and lipid ROS accumulation [43, 44]. Currently, the mechanism of ferroptosis involves iron metabolism, lipid metabolism, system Xc-GSH-GPX4, impaired mitochondrial energy metabolism, and other antioxidant pathways. The imbalance between these pathways will destroy the homeostasis of intracellular redox, produce excessive lipid peroxides and lipid ROS, and, thus, induce ferroptosis [45]. LDH is an important enzyme in the body that can affect energy metabolism. Its level increases in the myocardium and blood of myocardial infarction, myositis, and other diseases. The destruction of cell membrane structure caused by cell death or ferroptosis will also lead to the release of various enzymes such as cytoplasmic LDH [46]. The level of cytoplasmic LDH reflects the level of cell membrane permeability and cytotoxicity.

The expression of SLC7A11 can be induced under a variety of stress conditions, including oxidative stress, amino acid starvation, metabolic stress, and genotoxic stress. It may be an adaptive response that enables cells to restore redox homeostasis and maintain survival under stress conditions. Activating transcription factor 4 (ATF4) and/or Nrf2 are two major transcription factors that regulate stress-induced SLC7A11 expression [47]. By upregulating the expression of SLC7A11 through a variety of mechanisms, cancer cells enhance their antioxidant defense ability and inhibit ferroptosis [47]. Zhang et al. [48] indicated that SLC7A11-mediated cystine uptake not only promotes GSH synthesis but also GPX4 protein synthesis. Our study shows that SLC7A11 and GPX4 upregulated by Nrf2 in MI rats to inhibit ferroptosis. These results show that activation of Nrf2 might help to alleviate myocardial damage caused by myocardial infarction. We prophylactically upregulate miR-432-5 before inducing cardiac ischemia in rats, which alleviates the oxidative stress state of rats and reduces myocardial damage caused by ferroptosis.

On the other hand, we also noticed that the upregulated expression of Nrf2 under hypoxia/ischemia was sequential and the expression of Nrf2 reaches peak at 4–6 hr after H/R and then decreased. This shows that hypoxia is only effective in the upregulation and activation of Nrf2 in a certain time range, so the timing of upregulation of Nrf2 is also important. Our study shows that exogenous upregulation of miR-432-5p can downregulate the binding of Keap1 and Nrf2 *in vivo* and *in vitro*, thus indirectly upregulating the level of Nrf2.

## 5. Conclusion

In conclusion, we found that hypoxia and nonexogenous upregulation of miR-432-5p treatment can upregulate Nrf2 expression and further induce Nrf2 nuclear translocation, thus protect cardiomyocytes from ferroptosis caused by reperfusion and reduce the damage to cardiomyocytes. Our study also demonstrated that by binding Keap1, miR-432-5p indirectly activates Nrf2; of course, the activation effect of miR-432-5p needs to be further compared with the Nrf2 activator. Our study provides another idea that the exogenous upregulation of miR-432-5p can activate Nrf2 and finally slow down the myocardial injury caused by ferroptosis.

## Figures and Tables

**Table 1:** Sequence information.

Gene	Sequence
miR-432-5p NC	5′-CGAUCG CAU CAG CAU CGAUUG C-3′
miR-432-5p mimic	5′-UCUUGGAGUAGGUCAUUGGGUGG-3′
Keap1-WT	F: 5′-CCCAAGCTT ATGCAGCCAGATCCCAGGCC-3′
	R: 5′-CTAGTCTAGA TCAACAGGTACAGTTCTGCT-3′
Keap1-Mut	F: 5'-CCCAGGAAT ATGGACGGATAAGGCAGGCC-3'
	R: 5′-CTAGGCACCGTGCAGATGAAGAGATGTGCT-3′
nc-Nrf2	F: 5′-UUCUCCGAACGUGUCACGUTT-3′
	R: 5′-ACGUGACACGUUCGGAGAATT-3′
si-Nrf2 1	F: 5′-CCCUGUGUAAAGCUUUCAATT-3′
	R: 5′-UUGAAAGCUUUACACAGGGTT-3'
si-Nrf2 2	F: 5′-GGAGCAAGAUUUAGAUCAUTT-3′
	R: 5′-AUGAUCUAAAUCUUGCUCCTT-3′
si-Nrf2 3	F: 5′-GCUUCAGUGAUUCUGAAAUTT-3′
	R: 5′-AUUUCAGAAUCACUGAAGCTT-3′
miR-432-5p primer	F: 5′-AACGAGACGACGACAGACT-3′
	R: 5′-CTT GGAGTAGGTCAT TGG GT-3′
U6 primer	F: 5′-CTCGCTTCGGCAGCACA-3′
	R: 5′-CGCTTCACGAAT TTGCGTGTCAT-3′
GAPDH	F: 5′-AGAAGGCTGGGGCTCATTTG-3′
	R: 5′-AGGGGCCATCCACAGTCTTC-3′

**Table 2:** The heart function in rats from sham, NC-Lipo, and miR-432-5p-Lipo groups (*N* = 6 in each group).

	Sham		NC-Lipo		miR-432-5p-Lipo	
	Before anesthesia	4 hr After anesthesia	Before anesthesia	4 hr After anesthesia	Before anesthesia	4 hr After anesthesia
HR	391 ± 15	338 ± 21	382 ± 17	243 ± 23^*∗∗*^	402 ± 19	297 ± 22^*∗∗*^^, ##^
MAP (mm Hg)	66.27 ± 3.52	48.86 ± 3.84	67.58 ± 3.73	35.64 ± 4.02^*∗∗*^	65.88 ± 3.67	39.62 ± 4.13
LVDP (mm Hg)	123.4 ± 5.3	99.6 ± 7.6	116.8 ± 4.8	57.8 ± 11.4^*∗∗*^	112.7 ± 5.2	71.3 ± 10.3^*∗∗*^^, ##^
LVEDP (mm Hg)	5.95 ± 1.22	6.38 ± 1.25	6.11 ± 1.28	7.31 ± 1.29	6.06 ± 2.02	6.67 ± 2.11

HR, heart rate; MAP, mean arterial pressure; LVDP, left ventricular diastolic pressure; LVEDP, left ventricular end-diastolic pressure.  ^*∗∗*^compared to sham, *p* < 0.01 in same time point; ^##^compared to NO-Lipo, *p* < 0.01 in same time point.

## Data Availability

The datasets of GSE24548 and GSE31568 analyzed during the current study are available in the Gene Expression Omnibus (GEO) database (https://www.ncbi.nlm.nih.gov/geo/query/acc.cgi?acc=GSE24548 and https://www.ncbi.nlm.nih.gov/geo/query/acc.cgi).
